# Supplementary material for: Assessment of Comprehensive Patient-Reported Outcomes Before and After CPAP Therapy in Obstructive Sleep Apnea
Source: Biomedicines. 2025 Oct 27;13(11):2628. doi: 10.3390/biomedicines13112628 (PMC12649898; doi:10.3390/biomedicines13112628)
Supplement: Supplementary file 1 [file biomedicines-13-02628-s001.zip › biomedicines-3876832-supplementary.pdf]

Supplementary Table S1A. Multivariable Logistic Regression Predicting Improvement (Improved vs. Not Improved)

Outcome	Predictor	Adjusted OR	95% CI	p-value
**WHO Environment**	**CPAP adherence (%)**	**0.96**	**0.93–0.99**	**0.009**
**WHO Environment**	**Baseline score**	**0.89**	**0.82–0.97**	**0.010**
**WHO Social**	**Baseline score**	**0.87**	**0.80–0.95**	**0.001**

Supplementary Table S1B. Multivariable Linear Regression on Change Scores (Δ-Score, T2–T1)

Outcome	Predictor	β (Delta effect)	95% CI	p-value
**GAD-7**	**Baseline score**	**-0.35**	**-0.50–-0.20**	**0.000**
**GAD-7**	**CPAP adherence (%)**	**0.05**	**0.02–0.07**	**0.000**
**PHQ-9**	**CPAP adherence (%)**	**0.03**	**0.00–0.06**	**0.029**
**WHO Environment**	**Baseline score**	**-0.50**	**-0.67–-0.32**	**0.000**
**WHO Environment**	**CPAP adherence (%)**	**-0.15**	**-0.22–-0.07**	**0.000**
**WHO Physical**	**Baseline score**	**-0.45**	**-0.74–-0.15**	**0.004**
**WHO Psychological**	**Baseline score**	**-0.43**	**-0.64–-0.21**	**0.000**
**WHO Psychological**	**Sex (Female vs Male)**	**7.48**	**0.69–14.27**	**0.032**
**WHO Social**	**Baseline score**	**-0.45**	**-0.62–-0.28**	**0.000**
**WHO Social**	**CPAP adherence (%)**	**-0.10**	**-0.18–-0.03**	**0.006**
